# miR-139/PDE2A-Notch1 feedback circuit represses stemness of gliomas by inhibiting Wnt/β-catenin signaling

**DOI:** 10.7150/ijbs.62858

**Published:** 2021-08-12

**Authors:** San-Zhong Li, Kai-Xi Ren, Jing Zhao, Shuang Wu, Juan Li, Jian Zang, Zhou Fei, Jun-Long Zhao

**Affiliations:** 1 Department of Neurosurgery, Xijing Hospital, Fourth Military Medical University, Xi'an 710032, China.; 2 Department of Neurology, Tangdu Hospital, Fourth Military Medical University, Xi'an 710032, China.; 3 Department of Anesthesiology, Xijing Hospital, Fourth Military Medical University, Xi'an 710032, China.; 4 State Key Laboratory of Cancer Biology, Department of Medical Genetics and Developmental Biology, Fourth Military Medical University, Xi'an 710032, China.

**Keywords:** PDE2A, miR-139, glioma stem cell, Notch1, Wnt/β-catenin

## Abstract

**Rationale:** The malignant phenotypes of glioblastomas (GBMs) are primarily attributed to glioma stem cells (GSCs). Our previous study and other reports have suggested that both miR-139 and its host gene PDE2A are putative antitumor genes in various cancers. The aim of this study was to investigate the roles and mechanisms of miR-139/PDE2A in GSC modulation.

**Methods:** Clinical samples were used to determine miR-139/PDE2A expression. Patient-derived glioma stem-like cells (PD-GSCs) were stimulated for immunofluorescent staining, sphere formation assays and orthotopic GBM xenograft models. Bioinformatic analysis and further *in vitro* experiments demonstrated the downstream molecular mechanisms of miR-139 and PDE2A. OX26/CTX-conjugated PEGylated liposome (OCP) was constructed to deliver miR-139 or PDE2A into glioma tissue specifically.

**Results:** We demonstrated that miR-139 was concomitantly transcribed with its host gene PDE2A. Both PDE2A and miR-139 indicated better prognosis of gliomas and were inversely correlated with GSC stemness. PDE2A or miR-139 overexpression suppressed the stemness of PD-GSCs. FZD3 and β-catenin, which induced Wnt/β-catenin signaling activation, were identified as targets of miR-139 and mediated the effects of miR-139 on GSCs. Meanwhile, PDE2A suppressed Wnt/β-catenin signaling by inhibiting cAMP accumulation and GSK-3β phosphorylation, thereby modulating the self-renewal of PD-GSCs. Notably, Notch1, which is also a target of miR-139, suppressed PDE2A/miR-139 expression directly via downstream Hes1, indicating that miR-139 promoted its own expression by the miR-139-Notch1/Hes1 feedback circuit. Expectedly, targeted overexpression miR-139 or PDE2A in glioma with OCP system significantly repressed the stemness and decelerated glioma progression.

**Conclusions:** Our findings elaborate on the inhibitory functions of PDE2A and miR-139 on GSC stemness and tumorigenesis, which may provide new prognostic markers and therapeutic targets for GBMs.

## Introduction

Glioblastomas (GBMs) are the most lethal central nervous system (CNS) cancers, with an annual incidence of 50 per million individuals and median survival times of less than 15 months 1, 2. The features of invasive growth and resistance to conventional chemo- or radiotherapies make recurrence inevitable after surgical glioma resection. One of the major reasons for poor prognosis is glioma stem cells (GSCs) 3. GSCs are characterized by high expression of the stem cell markers CD133, Nestin and Sox2 and extensive self-renewal and multilineage differentiation potentials 3-5. Recent studies found that the initiation, progression, metastasis and recurrence of gliomas were mainly driven by GSCs 6-8. Understanding how GSCs are generated and how to accelerate differentiation to exhaust GSCs will contribute greatly to glioma treatment. Several transcription factors and signaling pathways, including Notch, Hedgehog and Wnt, are involved in GSC stemness modulation 9, 10. However, the detailed molecular mechanisms remain unclear. New effective therapeutics targeting GSCs are urgently needed.

MicroRNAs (miRNAs) are highly conserved small noncoding RNA molecules, 21-23 nucleotides in length. These molecules are generated through Dicer-mediated cleavage from pre-miRNAs and bind to 3'-UTR sequences through the specific recognition of miRNA seed sequences to further suppress target gene expression posttranscriptionally 11. miRNAs are ubiquitously expressed in eukaryotic organisms and participate in cell proliferation, apoptosis and differentiation 12, 13. Studies have reported that miRNAs play important roles in all stages of glioma tumorigenesis and progression, especially GSC development 14, 15. Due to their functional flexibility and diversity, miRNAs are considered diagnostic biomarkers and therapeutic targets. miR-139 has been reported to modulate the growth of different tumors and acts as an anti-oncogene 16, 17. miR-139 is located in the second intron of its host gene PDE2A. The levels of miR-139, PDE2A and DNA methylation in the PDE2A promoter were highly correlated in different tumor tissues according to the TCGA database, which suggested that miR-139 expression was dependent on PDE2A transcription 18, 19.

Previous studies have demonstrated that miR-139 is involved in glioma progression 20-22. However, whether miR-139 regulates GSCs has not been elucidated. In this study, we demonstrated that miR-139 and PDE2A expression was lower in GSCs. miR-139 obviously suppressed the self-renewal and promoted the differentiation of GSCs by inhibiting the specific targets FZD3 and β-catenin. Additionally, the overexpression of PDE2A also inhibited Wnt/β-catenin signaling through cAMP repression. In addition, Notch1, which was also demonstrated to be a target of miR-139, repressed the transcription of PDE2A and miR-139 via the Hes1 recognized site in the PDE2A promoter, suggesting that the miR-139-Notch1 double negative feedback loop could amplify the expression and function of PDE2A/miR-139. These results indicated that PDE2A/miR-139 could attenuate GSC activity and suppress glioma progression, which may provide novel strategies for glioma therapy.

## Materials and methods

### Human tissue samples

All human glioma tissues were obtained from patients undergoing surgery for glioma at the Department of Neurosurgery, Xijing Hospital, Fourth Military Medical University. The glioma samples were histologically classified by the diagnosis of clinical and pathological grading according to WHO guidelines. Written informed consent conforming to the tenets of the Declaration of Helsinki was obtained from each participant, and the study procedures were approved by the institutional review board of Fourth Military Medical University.

### Plasmid construction, cell culture and transfection

Fragments of the wild-type or mutated 3′-UTRs of FZD3, β-catenin and Notch1 were generated by PCR amplification from a human cDNA library and cloned into the pGL3-promoter plasmid (Promega, Madison, WI). The open reading frames (ORFs) of PDE2A, FZD3, β-catenin, N1ICD and constitutively activated β-catenin, which lost 90 amino acids at the N-terminus (Δ90-β-catenin), were amplified by using PCR and inserted into the eukaryotic expression vector pFlag-CMV (Invitrogen Life Technologies, Carlsbad, CA). Oligonucleotides were chemically synthesized and used at a final concentration of 50 nmol/L according to the manufacturer's instructions (RiboBio, Guangzhou, China). The sequences of oligonucleotides were as follows: shPDE2A, 5'-gctgttgtccaattcctcagg; si-Notch1 #1, 5'-gattgtccaggaaacaactgcaaga; and si-Notch1 #2, 5'-ccagagtggacaggtcagtactgta.

Human glioma cells were isolated from GBM patient #1 (glioma cell #1, GC#1), patient #2 (glioma cell #2, GC#2) and patient #3 (glioma cell #3, GC#3). GC#1, #2 and #3 were stimulated to transform into glioma stem cells by culturing in serum-free Dulbecco's modified Eagle's medium (DMEM)/F12 medium (Invitrogen Life Technologies) containing 20 ng/ml bFGF (human recombinant, Sigma-Aldrich, St. Louis, MO), 20 ng/ml EGF (mouse submaxillary, Sigma-Aldrich), N-2 supplement (Sigma-Aldrich), penicillin and streptomycin. The maintained GSCs were cultured in complete medium supplemented with 20% fetal bovine serum (FBS) and 2 mM glutamine (Invitrogen Life Technologies) for differentiation culture. In some cases, gamma secretase inhibitor (GSI) (Sigma-Aldrich) was added to the culture medium. The prepared cells were seeded onto 12-well or 6-well plates overnight and infected with different lentiviruses to overexpress PDE2A, FZD3, β-catenin, N1ICD or activated β-catenin (Δβ-cat). After infection, glioma cells were cultured in complete DMEM for further experiments. All cells were incubated at 37 °C in an atmosphere of 5% CO_2_.

### The synthesis of OX26/CTX-PL-DNA carrier

The glioma targeted DNA deliver system, which was designed as OX26/CTX-PL (OCP), was prepared and synthesized as described previously^23^. Briefly, Pegylated liposomes (PLs) and plasmid DNA (vehicle, pPre-miR-139 and pPDE2A) were mixed to form the unmodified PL, PL/pPre-miR-139 and PL/pPDE2A. The OX26 and CTX were thiolated by 2-iminothiolane at room temperature (RT) for 1 hour, and then were incubated with PL, PL/pPre-miR-139 and PL/pPDE2A overnight at RT. The reaction mixture was purified with a Sepharose column and the liposome complexes were eluted by PBS buffer to obtain OX26/CTX-PL (OCP), OX26/CTX-PL/pPre-miR-139 (OCP-miR-139) and OX26/CTX-PL/pPDE2A (OCP-PDE2A).

### Patient-derived xenograft (PDX) models

Eight-week-old nude mice (male BALB/cA-nu) were purchased from the Shanghai Experimental Animal Center (Chinese Academy of Sciences, Shanghai, China) and maintained under specific pathogen-free conditions. Fifteen mice were randomly divided into three groups. Glioma cells isolated from GBM patient #1 were stimulated to transform into glioma stem cells. The patient-derived GSCs were overexpressed luciferase by lentivirus and were then injected intracranially into each mouse at 1 × 10^6^ cells. Three days after inoculation, the mice were administered with OCP, OCP-miR-139 (10 μg plasmid DNA) or OCP-PDE2A (10 μg plasmid DNA) complexes through tail vein injection, respectively. The treatment was made once a week and the glioma growth was evaluated by bioluminescence imaging three weeks after the administration. Part of the tumor-bearing mice were sacrificed, and the brain tissues were excised, sectioned and frozen in liquid nitrogen for further analyses. For some experiment, the survival time of these mice was monitored for Kaplan-Meier survival curve analysis. All animal experiments were approved by the Animal Experiment Administration Committee of the Fourth Military Medical University. All methods were carried out in accordance with the recommendations of the Guide for the Care and Use of Laboratory Animals prepared by the National Academy of Sciences and published by the National Institutes of Health.

### RNA extraction and quantification assay

Total RNA was extracted from human glioma specimens or cultured cell samples with TRIzol reagent (Invitrogen) according to the manufacturer's instructions. Complementary DNA was prepared with a reverse transcription kit (Takara, Dalian, China) to detect the expression levels of stem cell markers, PDE2A, miR-139 and its downstream target. Quantitative real-time PCR was performed using a SYBR Premix EX Taq kit (Takara) and the ABI PRISM 7500 Real-time PCR system in triplicate, with GAPDH or U6 snRNA as the internal control. Primers are listed as follows: CD133 forward, 5'-agtcggaaactggcagatagc and reverse, 5'-ggtagtgttgtactgggccaat; Nestin forward, 5'-ctgctacccttgagacacctg and reverse, 5'-gggctctgatctctgcatctac; SOX2 forward, 5'-gccgagtggaaacttttgtcg and reverse, 5'-ggcagcgtgtacttatccttct; FZD3 forward, 5'-gttcatggggcatataggtgg and reverse, 5'-gctgctgtctgttggtcataa; β-catenin forward, 5'-aaagcggctgttagtcactgg and reverse, 5'-cgagtcattgcatactgtccat; Notch1 forward, 5'-gaggcgtggcagactatgc and reverse, 5'-cttgtactccgtcagcgtga; PDE2A forward, 5'-gaaagtccgggaggctatcat and reverse, 5'-cacttgggtatcaggagcca; Axin2 forward, 5'-caacaccaggcggaacgaa and reverse, 5'-gcccaataaggagtgtaaggact; CyclinD1 forward, 5'-gctgcgaagtggaaaccatc and reverse, 5'-cctccttctgcacacatttgaa; c-Myc forward, 5'-ggctcctggcaaaaggtca and reverse, 5'-ctgcgtagttgtgctgatgt; and GAPDH forward, 5'-ggagcgagatccctccaaaat and reverse, 5'-ggctgttgtcatacttctcatgg. To determine the level of miR-139, the Bulge-Loop miRNA qRT-PCR Detection Kit (RiboBio) was utilized according to the manufacturer's instructions. RNA expression was normalized to the level of human U6 snRNA.

### Western blotting

Cells were harvested and lysed on ice for 30 min in RIPA buffer supplemented with protease inhibitors (100 mM Tris-HCl, pH 7.4, 150 mM NaCl, 5 mM EDTA, 1% Triton X-100, 1% deoxycholate acid, 0.1% SDS, 2 mM phenylmethylsulfonyl fluoride, 1 mM sodium orthovanadate, 2 mM DTT, 2 mM leupeptin, 2 mM pepstatin). The lysates were centrifuged, and the supernatants were collected as total proteins. Equal amounts of each protein sample were separated by SDS-PAGE and transferred onto PVDF membranes after the concentrations were detected by using a BCA kit (Beyotime, Haimen, China). After blocking with 5% nonfat dried milk solution for 2 h, the membranes were incubated with primary antibodies against FZD3 (polyclonal, Sigma-Aldrich), β-catenin (15B8, Sigma-Aldrich), Notch1 (mN1A, Sigma-Aldrich), GSK3β (ab131356, Abcam, Cambridge, MA), phosphorylated GSK3β (ab75745, Abcam), PDE2A (ab224616, Abcam) and β-actin (Boster Bio Tec, Wuhan, China). After washing three times with PBST, the membranes were then incubated with HRP-conjugated secondary antibody and visualized with an ECL detection system.

### Luciferase reporter assay

For the target gene validation experiments, wild-type or mutated 3′-UTRs of targets were inserted downstream of the luciferase ORF region to construct a reporter assay system. For the transcription modulation experiments, the enhancer region of PDE2A was cloned upstream of the luciferase ORF region to construct a reporter assay system. For the Wnt/β-catenin activation experiments, the β-catenin/TCF recognized motif was inserted upstream of the luciferase ORF region to construct a reporter assay system. HEK293T cells were plated in 96-well plates in advance and cotransfected with the reporter assay system and pRL-TK vector, as well as miR-139 oligonucleotides or N1ICD plasmid, respectively. The cells were harvested and lysed with lysis buffer 24 h later (Promega, Madison, WI). The relative luciferase activity was determined using the Dual Luciferase Reporter Assay System (Promega, Madison, WI, USA), and firefly luciferase activity was normalized to the relative activity of Renilla luciferase. Each experiment was performed at least five times, and the data were analyzed with Student's t-test.

### ChIP assay

The chromatin immunoprecipitation (ChIP) assay was performed using a kit (Merck Millipore) according to the manufacturer's instructions. Glioma stem cells were fixed with formaldehyde. The cross-linked immune complexes were further sonicated into DNA fragments of 200-500 bp in length and precipitated with anti-Hes1 antibody. DNA was extracted from the collected samples and analyzed by PCR with the following primers: Site1 forward, 5'-agagtccaaatctctcctt and reverse, 5'-tacttgaagagaaaccaga and Site2 forward, 5'-cttgagatagggaagagt and reverse, 5'-taatcccactctcgatgac.

### Sphere formation assay

Single-cell suspensions of GSCs were cultured in serum-free DMEM/F12 medium for 7 days. The number of GSC spheres was counted under a microscope to assess the self-renewal ability. For the detection of gene expression, GSC spheres were harvested on the 5^th^ day and dissociated mechanically into a single-cell suspension for RNA extraction and other experiments. For the differentiation of GSC spheres, tertiary spheres were plated onto polylysine (Sigma-Aldrich)-coated coverslips in DMEM/F12 medium with 20% fetal bovine serum to allow sphere differentiation. At different times of differentiation, GSC spheres were harvested for further detection.

### Measurement of cAMP concentration

For cAMP measurement, GSCs under different treatments or stimulations were immediately frozen in liquid nitrogen and lyophilized at -80 °C for 24 h. The lyophilized GSCs were weighed, and their cAMP contents were measured using a cAMP EIA kit (Cayman Chemical). The cAMP content is expressed as picomoles per gram of dry tissue weight.

### Cell cycle detection

The cell cycle distribution was determined by using a BD Calibur Flow Cytometer (BD, Franklin Lakes, NJ). Briefly, the cells were collected and fixed in ice-cold ethanol (70% in PBS) overnight at 4 °C. The cells were treated with 20 g/ml RNase A (Sigma-Aldrich) for 1 h at 37 °C to degrade the RNA and incubated with 50 μg/ml propidium iodide (Sigma-Aldrich) in the dark. The DNA content was analyzed by flow cytometry, and all phases of the cell cycle were analyzed by the appropriate gating on the distribution plot.

### Proliferation assays

The proliferation of glioma cells was analyzed with CCK8 assay. Briefly, glioma cells overexpressing miR-139 or PDE2A were seeded into 96-well plates with different treatments, and evaluated cell proliferation at 24 h, 48 h, 72 h and 96 h using the CCK8 reagent Kit. After the incubation for 4 h at 37 °C, the supernatant was removed and the precipitation was dissolved in DMSO (Sigma). Spectrophotometric absorbance was measured at the wavelength of 490 nm by a microplate reader (BioTek Instruments Inc., Winooski, VT).

### Immunofluorescence staining

The tumor tissues of mice and clinical specimens were fixed with 4% paraformaldehyde, followed by antigen retrieval. After blocking with 5% BSA, the sections were incubated with different antibodies, followed by staining with 4',6-diamidino-2-phenylindole (DAPI). Slices of normal, adjacent and glioma tissues from patients were subjected to miR-139 hybridization *in situ* (Servicebio, Wuhan, China) followed by PDE2A staining (Abcam). The mouse tumor tissues were stained for Ki67 or CD31 (BioLegend, San Diego, CA). For GSC identification, the cells were stained for Nestin, GFAP, MAP2 and OLIG2 (Abcam). The stained samples were observed under a laser scanning confocal microscope (FV-1000, Olympus, Tokyo, Japan). The data were analyzed with FlowJo vX.0.6 software (FlowJo, LLC, Ashland, OR).

### Statistical analysis

The experiments were repeated independently at least three times, and the data are presented as the means ± standard deviation. Student's t-test or one-way ANOVA with Tukey's multiple comparison test was performed for comparisons between groups. The survival time was analyzed by using Kaplan-Meier survival analysis. The statistical results are expressed as the means ± SD. P<0.05 was considered significant, and P<0.01 was highly significant.

## Results

### PDE2A and miR-139 were coexpressed in human gliomas and negatively correlated with the stemness and progression of gliomas

**Figure 1:**
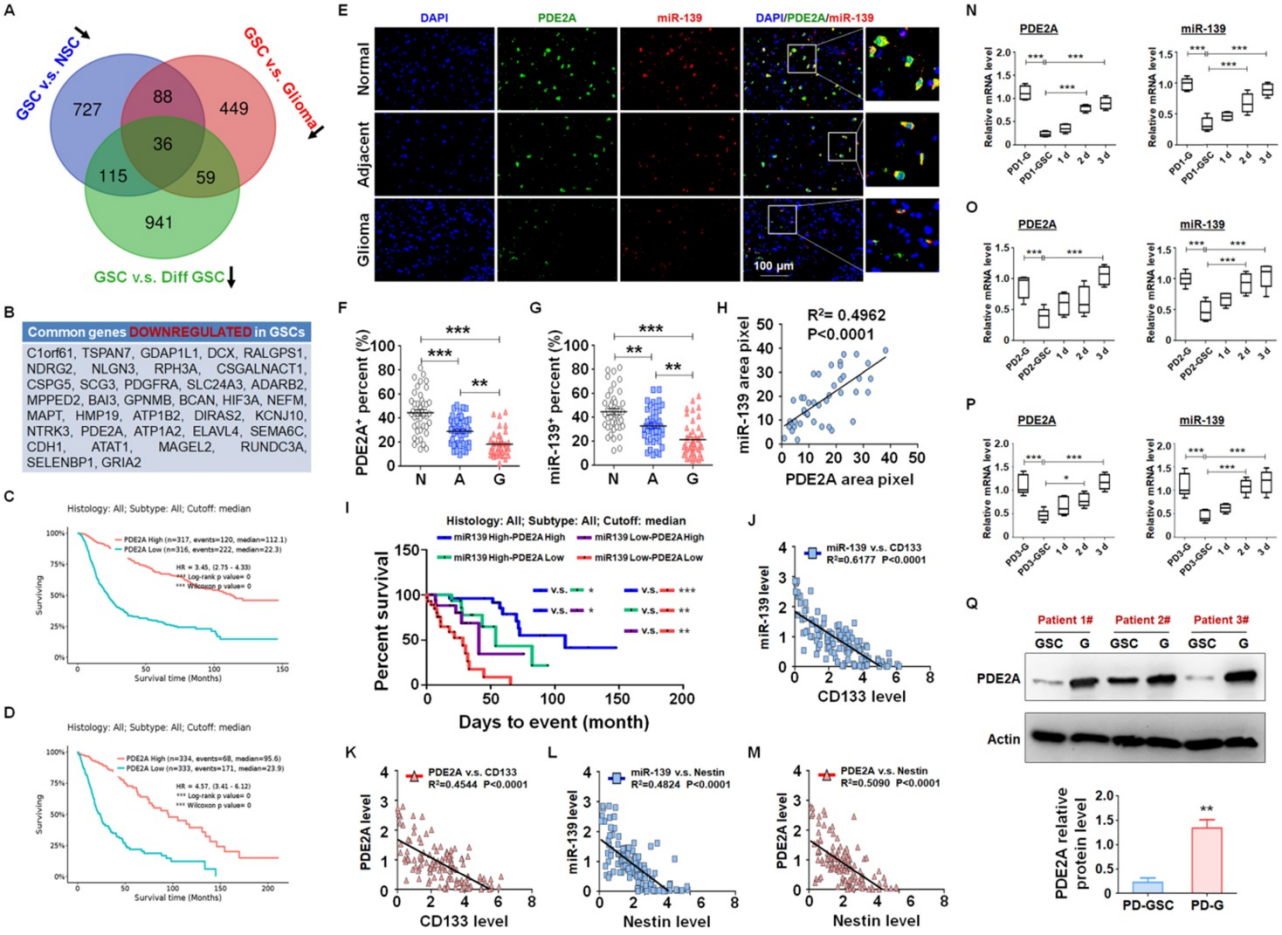
** PDE2A and miR-139 were coexpressed in human gliomas and negatively correlated with stemness of gliomas. (A-B)** The differential mRNA expression profiles from public data between GSCs and NSCs (GSE41033), GSCs and glioma cells (GSE124145) and GSCs and differentiated GSCs (GSE68343) were analyzed. The three sets of expression data were compared, and the common downregulated genes in GSCs are presented in a Venn diagram (A) and list (B). **(C-D)** The correlation between PDE2A expression and survival time of glioma patients was analyzed from the CGGA (C) and TCGA (D) databases. **(E-G)** The expression of PDE2A and miR-139 was examined in normal (n = 39), adjacent (n = 39) and glioma (n = 39) tissues from glioma patients using immunofluorescence staining followed by *in situ* hybridization. The percentages of PDE2A^+^ (F) and miR-139^+^ (G) cells were quantitatively compared. **(H)** The expression correlation between PDE2A and miR-139 was analyzed by staining density (n = 39). **(I)** Kaplan-Meier survival analysis of glioma patients is represented according to the expression levels of PDE2A and miR-139 (n = 125). **(J-M)** The relationship between PDE2A/miR-139 and CD133 (J-K) or Nestin (L-M) expression in glioma tissues was assessed (n = 125). **(N-P)** Glioma cells derived from glioma patients (PD-G) were stimulated to transform into GSCs (PD-GSCs) and then cultured in complete medium for differentiation. The expression of PDE2A and miR-139 was detected in PD-G, maintained PD-GSCs and differentiated PD-GSCs (n = 5). **(Q)** The protein level of PDE2A was examined by WB in PD-GSCs and PD-G (n = 3). Bars, means ± SEM; *, P < 0.05; **, P < 0.01; ***, P < 0.001.

miRNAs are pivotal in glioma progression, especially in the maintenance of GSC stemness. The expression of miRNAs is regulated at the level of primary transcripts or their host genes. Generally, miRNAs and host genes play cooperative roles in biological processes. To identify the key host gene/miRNA pairs involved in GSC regulation, we analyzed the published differential mRNA expression profiles between GSCs and NSCs (GSE41033) 24, GSCs and glioma cells (GSE124145) and GSCs and differentiated GSCs (GSE68343) (Fig. S1). After comparing the three sets of sequencing data, we identified 27 GSC suppressors that were downregulated in GSCs (Fig. 1A-B) and predicted a better prognosis of glioma patients from the TCGA database (Fig. 1C-D and S2). Among these GSC suppressors, PDE2A was the host gene of miR-139, which was also downregulated in GSCs (Fig. S3) and inhibited glioma progression 21.

miR-139 located in the second intron of PDE2A both in human and mouse genomes. PDE2A immunofluorescence staining combined with miR-139 *in situ* hybridization was performed in normal, adjacent and glioma sections from glioma patients (Fig. 1E). The PDE2A- or miR-139-positive rate in normal tissue was much higher than that in adjacent tumors or gliomas (Fig. 1F-G). In addition, the fluorescence intensity of miR-139 was highly correlated with that of PDE2A in each single cell (Fig. 1H). Furthermore, the mRNA levels of PDE2A and miR-139 in glioma tissues were consistent (Fig. S4). We further analyzed the relationship between PDE2A and miR-139 expression and glioma patient survival time. The results suggested that patients with high expression of both PDE2A and miR-139 presented the best prognosis, and lower levels of both genes predicted a poor prognosis for gliomas (Fig. 1I).

Considering that PDE2A and miR-139 were inhibited in GSCs, the relationship of PDE2A and miR-139 with GSC stemness was determined. The results showed that PDE2A and miR-139 both shared a completely inverse expression tendency with the GSC markers CD133 (Fig. 1J-K) and Nestin (Fig. 1L-M). Next, glioma cells were isolated from three GBM patients, and patient-derived glioma cells (PD-G) were stimulated into glioma stem cells (PD-GSCs), which were identified by Nestin staining (Fig. S5A) and pluripotent differentiation ability after culture in complete medium containing FBS (Fig. S5B and S5C). The expression levels of CD133, Nestin and SOX2 were determined in PD-G, PD-GSCs and differentiated PD-GSCs, and the results indicated that stemness was increased in PD-GSCs and attenuated during differentiation in all patients (Fig. S5D-F). We further detected PDE2A and miR-139 expression in PD-G and treated PD-GSCs. As expected, miR-139 and PDE2A, sharing the opposite expression tendency with stemness markers, decreased in PD-GSCs and recovered as differentiated (Fig. 1N-P). The protein analysis of PDE2A resulted in the same conclusion (Fig. 1Q). These results indicated that miR-139 and its host gene PDE2A were involved in the regulation of GSC stemness.

### Both PDE2A and miR-139 reduced the stemness of GSCs

**Figure 2:**
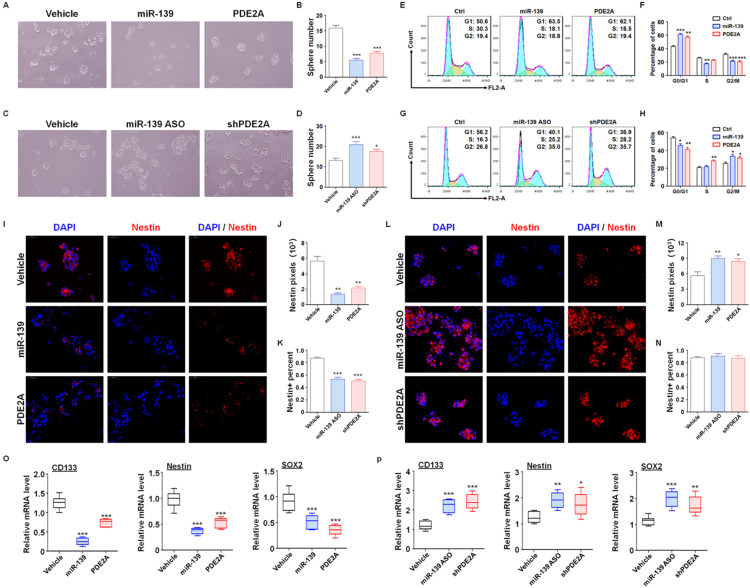
** PDE2A and miR-139 reduced the self-renewal and promoted the differentiation of GSCs. (A-B)** The sphere formation of PD-GSCs was analyzed when miR-139, PDE2A or vehicle was transfected by lentivirus (n = 6). **(C-D)** The sphere formation of PD-GSCs was analyzed when miR-139 and PDE2A were inhibited by ASO and shRNA, respectively (n = 6). **(E-F)** PD-GSCs were treated as described in (A), and the cell cycle was evaluated (n = 6). **(G-H)** PD-GSCs were treated as described in (C), and the cell cycle was evaluated (n = 6). **(I-K)** PD-GSCs overexpressing miR-139 or PDE2A and Nestin were analyzed by immunofluorescence staining (I). Nestin expression (J) and Nestin-positive cell percentages (K) were determined (n = 6). **(L-N)** miR-139 and PDE2A were inhibited by ASO and shRNA in PD-GSCs, and Nestin was analyzed by immunofluorescence staining (L). Nestin expression (M) and Nestin-positive cell percentages (N) were determined (n = 6). **(O-P)** The expression of stemness markers was determined by RT-PCR when miR-139 and PDE2A were overexpressed (O) (n = 6) or inhibited (P) (n = 6) in PD-GSCs. Bars, means ± SEM; **, P < 0.01; ***, P < 0.001.

Next, further assessments were performed to verify the regulatory effect of miR-139 and PDE2A on GSC functions. The *in vitro* experiments suggested that overexpression of PDE2A and miR-139 obviously attenuated the GSC sphere formation ability (Fig. 2A-B and Fig. S6). Inhibiting PDE2A by shRNA and blocking miR-139 by ASO both increased the numbers of GSC spheres (Fig. 2C-D). The further experiments also indicated that PDE2A and miR-139 arrested the cell cycle of GSCs at G1 phase (Fig. 2E-F) and decreased the proliferation of GSCs (Fig. 2G). On the other hand, repression of PDE2A or miR-139 accelerated the cell cycle and proliferation ability of GSCs (Fig. 2H-J).

We also evaluated the regulatory effect of PDE2A and miR-139 on GSC stemness maintenance. PDE2A and miR-139 overexpression reduced both Nestin expression and Nestin+ cell percentages in GSCs (Fig. 2K-M). Moreover, when PDE2A or miR-139 was repressed, although the Nestin+ cell percentage remained unchanged, Nestin expression was increased (Fig. 2N-P). Moreover, the mRNA expression of stemness markers was repressed by miR-139 and PDE2A (Fig. 2Q-R). These results suggested that both PDE2A and miR-139 decreased the self-renewal and stemness maintenance of GSCs *in vitro*.

### miR-139 suppressed stemness maintenance by inhibiting Wnt/β-catenin signaling in GSCs

**Figure 3:**
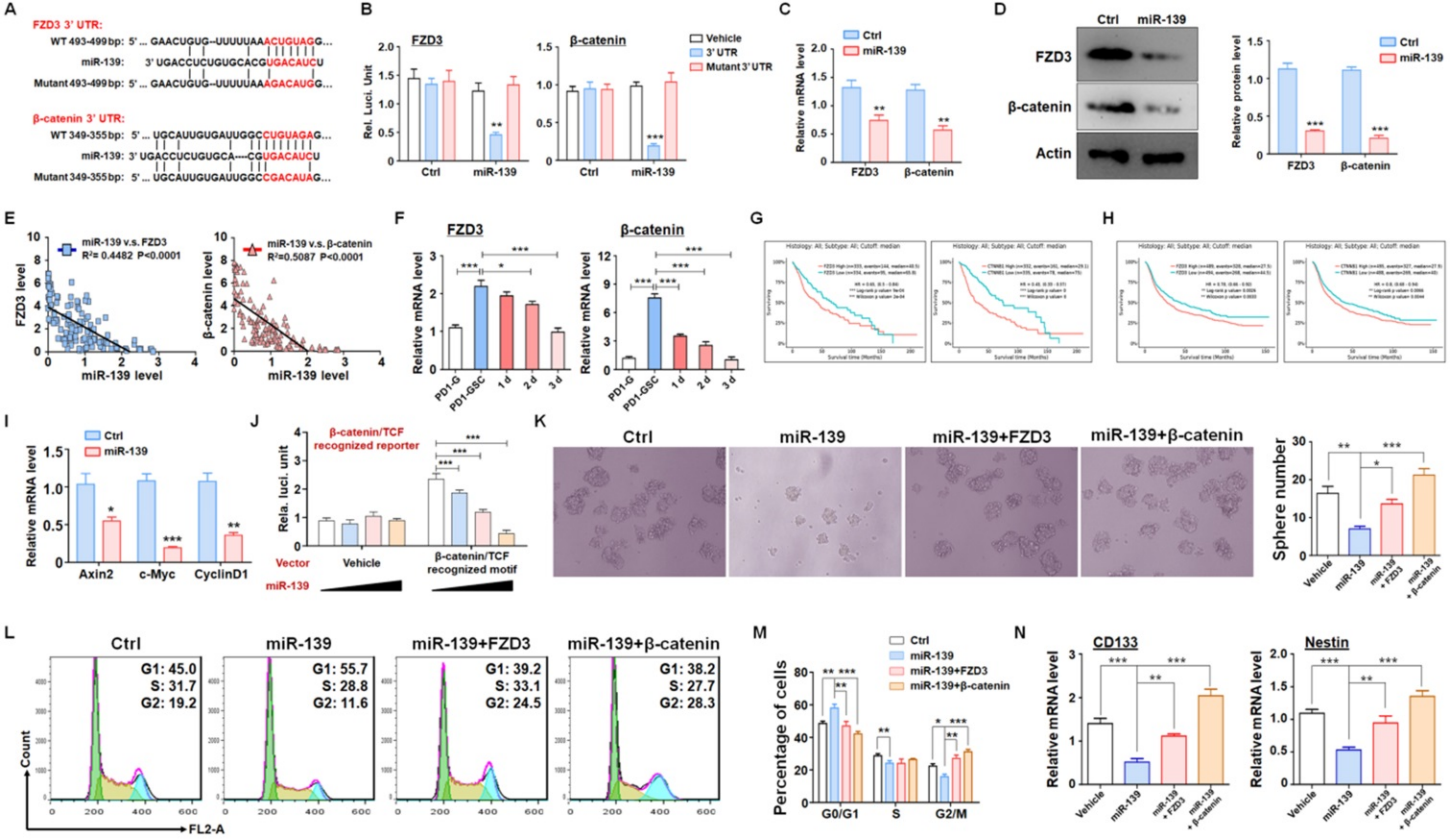
** FZD3 and β-catenin were validated as targets of miR-139 in gliomas and regulated GSC stemness. (A-B)** Bioinformatic prediction identified FZD3 and β-catenin as targets of miR-139 (A), and a reporter assay was carried out to validate the results (B) (n = 6). **(C-D)** The mRNA levels (C) and protein expression (D) of FZD3 and β-catenin were detected in miR-139-overexpressing PD-GSCs (n = 6). **(E)** The correlations between the expression levels of miR-139 and the target genes were analyzed in human glioma tissues (n = 125). **(F)** The expression of FZD3 and β-catenin was detected in glioma cells, maintained GSCs and differentiated GSCs (n = 6). **(G-H)** Kaplan-Meier survival analysis of glioma patients is represented according to the expression levels of FZD3 or β-catenin from the TCGA (G) and CGGA (H) databases. **(I)** The downstream molecules of Wnt/β-catenin signaling were determined by RT-PCR in PD-GSCs overexpressing miR-139 or vehicle (n = 6). **(J)** The activation of Wnt/β-catenin signaling was detected by reporter assay after miR-139 overexpression (n = 6). **(K)** PD-GSCs were infected with a lentivirus expressing miR-139 alone or coexpressing target genes. The sphere formation assay was performed in these groups (n = 6). **(L-M)** PD-GSCs were treated as described in (K), and the cell cycle was detected in different groups (n = 6). **(N)** The expression of stemness markers was detected in PD-GSCs treated the same as in (K) (n = 6). Bars, means ± SEM; *, P < 0.05; **, P < 0.01; ***, P < 0.001.

To fully understand the molecular mechanisms, we predicted targets of miR-139 by several bioinformatic algorithms (PicTar, TargetScan and miRDB), and FZD3 and β-catenin were identified as candidates (Fig. 3A). FZD3, which is a membrane receptor of Wnts ligands, belongs to the Frizzleds family and induces Wnt/β-catenin signaling 25. Several studies have reported that Wnt/β-catenin signaling is required for cell proliferation and plays a significant role in GSC generation, differentiation and self-renewal 26. The luciferase reporter assay showed that miR-139 suppressed the luciferase of the reporter plasmid containing the wild-type FZD3 or β-catenin 3'-UTR, and disruption of the seed sequence in the FZD3 or β-catenin 3'-UTR abrogated this effect (Fig. 3B). In addition, the mRNA levels of targets were determined in miR-139-overexpressing glioma cells, which showed that miR-139 inhibited FZD3 and β-catenin expression (Fig. 3C). A similar conclusion was also obtained by protein level examination (Fig. 3D). We also analyzed the relationship between the expression levels of the two target genes and miR-139 in human glioma tissues. The data showed that both FZD3 and β-catenin presented an inverse correlation with miR-139 (Fig. 3E). The further detection demonstrated that the expression of FZD3 and β-catenin was upregulated in GSC and decreased gradually as differentiation, which is opposite with miR-139 (Fig. 3F). The Kaplan-Meier survival analysis of glioma patients from the TCGA (Fig. 3G) and CGGA (Fig. 3H) databases indicated that lower expression of FZD3 and β-catenin predicted better prognosis. Moreover, the activation of Wnt/β-catenin signaling was assessed by RT-PCR and reporter assay. The expression of downstream molecules, including Axin2, c-Myc and CyclinD1, was all repressed after miR-139 overexpression (Fig. 3I). And the activity of β-catenin/TCF complex was also repressed by miR-139 (Fig. 3J). These results indicated that miR-139 obviously reduced the expression of FZD3 and β-catenin and the subsequent Wnt signaling activation.

To confirm that the function of miR-139 in GSC regulation was based on FZD3 and β-catenin reduction, GSCs overexpressing miR-139 were transfected with a FZD3 or β-catenin expression lentivirus, and sphere formation was determined. Rescuing FZD3 or β-catenin completely restored the colony sphere formation ability of GSCs (Fig. 3K). The cell cycle of GSCs was also restored after FZD3 or β-catenin overexpression (Fig. 3L-M). In addition, the expression of the GSC markers CD133 and Nestin was induced by both targets of miR-139 (Fig. 3N). These data suggested that FZD3 and β-catenin, as target genes of miR-139, promoted GSC stemness maintenance and completely rescued the effect of miR-139 on GSC regulation.

### PDE2A decreased cAMP generation and suppressed Wnt/β-catenin signaling to regulate GSC stemness

**Figure 4:**
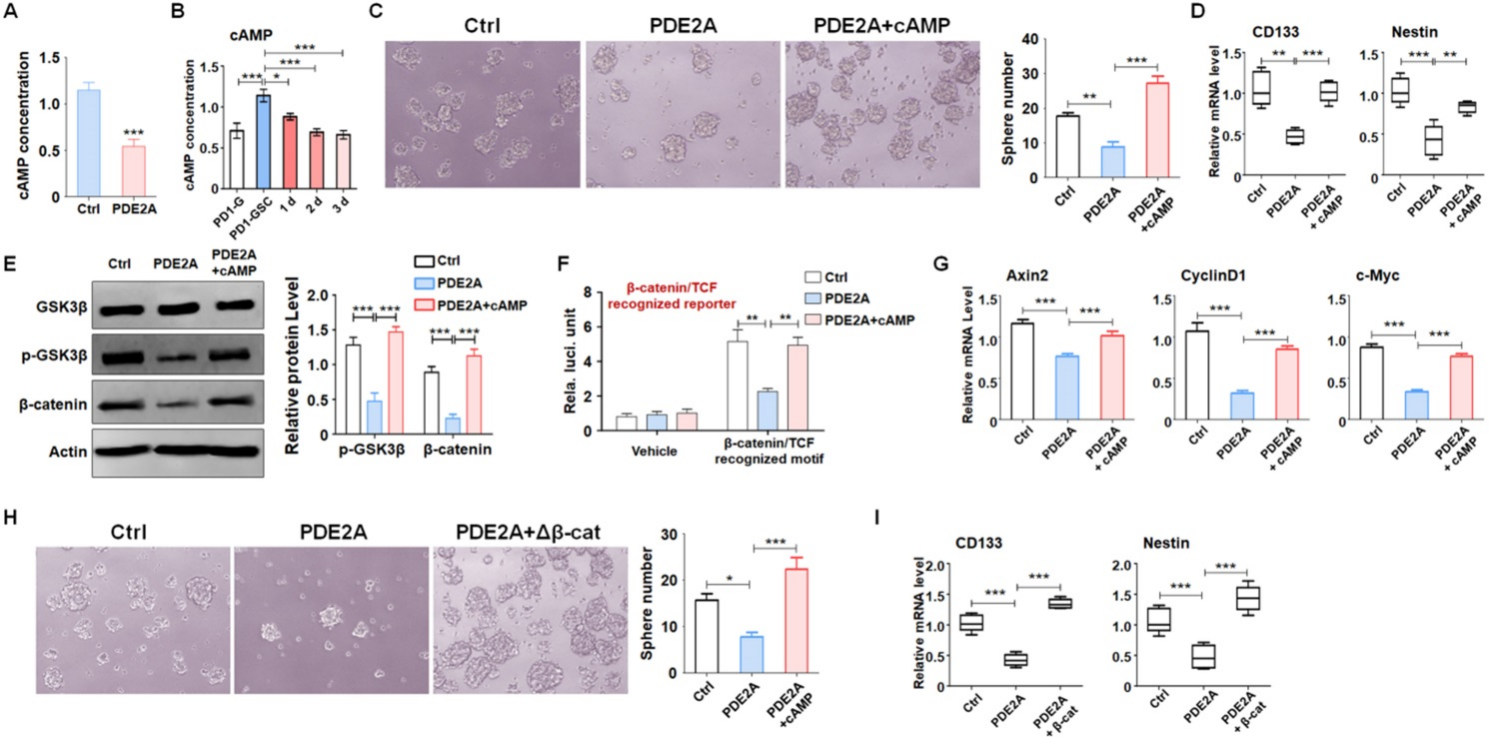
** PDE2A regulated GSC stemness by inhibiting cAMP generation. (A)** The concentration of cAMP in gliomas was examined when PDE2A was overexpressed (n = 6). **(B)** cAMP concentration was measured in glioma cells, maintained GSCs and differentiated GSCs (n = 6). **(C)** The self-renewal ability was determined by sphere formation assay when PD-GSCs were infected with vehicle, PDE2A or PDE2A in the presence of cAMP (20 nM) (n = 6). **(D)** The expression of CD133 and Nestin was detected in GSCs treated as shown in (C) (n = 6). **(E)** PD-GSCs were treated as in (C), and GSK3β phosphorylation and β-catenin expression were determined by WB (n = 5). **(F)** The glioma cells were transfected with TCF/LEF reporter system or vehicle, as well as PDE2A overexpression or/and additional cAMP. Then the activation of TCF/LEF reporter was measured by reporter assays (n = 6). **(G)** PD-GSCs were treated as in (C), and the mRNA levels of Wnt downstream molecules were determined by RT-PCR (n = 6). **(H-I)** PDE2A alone or accompanied by activated β-catenin was overexpressed in PD-GSCs. The sphere formation ability (H) (n = 6) and stemness marker expression (I) (n = 6) were evaluated in different groups. Bars, means ± SEM; *, P < 0.05; **, P < 0.01; ***, P < 0.001.

Increasing evidence has demonstrated that activated PDE2A hydrolyzes cAMP and terminates signaling delivery 27. The balance of the cAMP concentration plays an important role in central nervous system development 28, 29. We examined the concentration of cAMP in gliomas when PDE2A was overexpressed and found that PDE2A greatly reduced cAMP generation (Fig. 4A). In addition, cAMP levels were higher in GSCs than in differentiated glioma cells (Fig. 4B). Further experiments showed that supplementation of cAMP restored GSC self-renewal (Fig. 4C) and stemness, even as PDE2A was overexpressed (Fig. 4D).

The elevation of intracellular cAMP induces the accumulation of PKA and further activates the Wnt signaling pathway through a phosphorylation cascade 30. Western blotting showed that PDE2A suppressed GSK3β phosphorylation and β-catenin protein levels, which could be rescued by exogenous cAMP administration (Fig. 4E). In addition, the activity of Wnt/β-catenin signaling was evaluated by TCF/LEF reporter assays. The results displayed that the activation of TCF/LEF reporter system was decreased when PDE2A was overexpressed and promoted by exogenous cAMP (Fig. 4F). The expression levels of Axin2, CyclinD1 and c-Myc, which are downstream molecules of Wnt/β-catenin signaling, were also reduced by PDE2A overexpression. However, additional cAMP completely recovered Wnt signaling activation (Fig. 4G). Considering that the autocrine of Wnt signaling is one of characteristics of cancer stemness, the secretion of Wnt ligands was determined after miR-139/PDE2A overexpression. The results indicated that miR-139 or PDE2A did not influence the expression of classical Wnt ligands (Fig. S7).

To evaluate the regulation of Wnt/β-catenin signaling on GSC stemness, we packaged a lentivirus containing a constitutively activated β-catenin with a 90 aa depletion in the N-terminus and overexpressed this Δβ-catenin protein in GSCs to induce Wnt signaling. The results showed that forced activation of Wnt signaling could rescue the effects of proliferation blockade (Fig. 4H) and stemness loss mediated by PDE2A on GSCs (Fig. 4I). The above data demonstrated that PDE2A regulated GSC development by inhibiting the cAMP-β-catenin axis.

### miR-139 amplified its own expression through the double negative feedback loop of miR-139-Notch1 regulation

**Figure 5:**
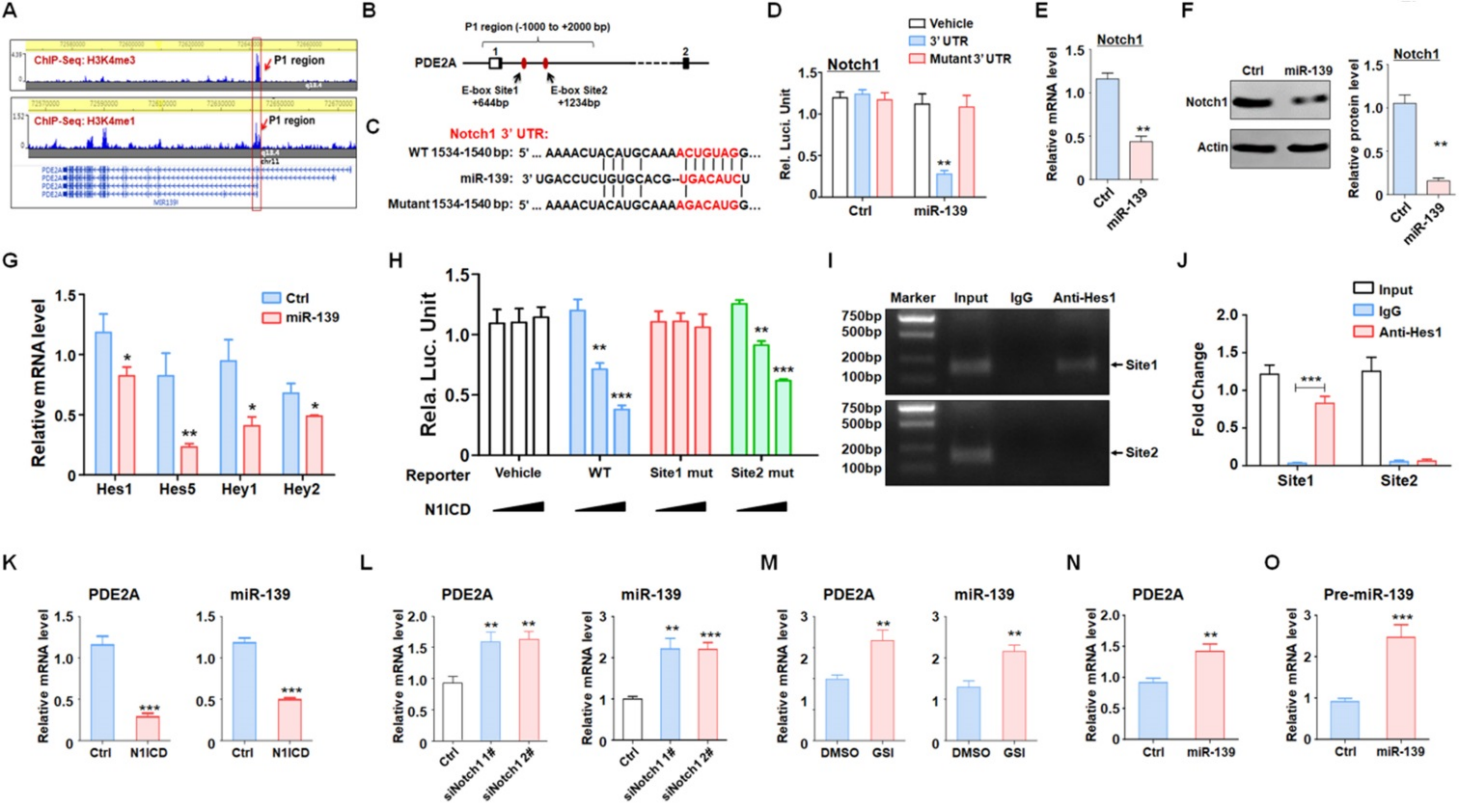
** miR-139 amplified its own expression through the double negative feedback loop of miR-139-Notch1 regulation. (A)** The promoter and enhancer distribution near the PDE2A/miR-139 gene loci. A strong promoter and enhancer region (P1) was found. **(B)** The P1 region harbored two Hes1 recognition sites. **(C-D)** Bioinformatic prediction identified Notch1 as another target of miR-139 (C), and a reporter assay was carried out to validate the results (D) (n = 6). **(E-F)** The mRNA levels (E) and protein expression (F) of Notch1 were detected in miR-139-overexpressing PD-GSCs (n = 6). **(G)** The downstream molecules of Notch signaling were detected by RT-PCR in PD-GSCs overexpressing miR-139 or vehicle (n = 6). **(H)** Reporter assays were performed to evaluate the regulation of the P1 region mediated by Notch (n = 6). **(I-J)** The ChIP assay results validated the Notch signaling-modulated P1 region transcription activity (n = 5). **(K-M)** PDE2A and miR-139 expression was determined when Notch signaling was activated (K) (n = 6) or inhibited by siRNA (L) (n = 6) or inhibitor (GSI) (M) (n = 6). **(N-O)** The expression of PDE2A and pre-miR-139 was detected when miR-139 was overexpressed (n = 6). Bars, means ± SEM; **, P < 0.01; ***, P < 0.001.

Several studies and our present data (Fig. 1) demonstrated that miR-139 expression was correlated with that of its host gene. We evaluated the histone activation of the PDE2A promoter region in the brain from the cistrome database (cistrome.org) 31, 32. PDE2A possessed a unique promoter region (H3K4me3 peak, P1) and several enhancer regions (H3K4me1 peaks), which indicated that promoter P1 shared strong enhancer activity and was responsible for the transcription of different isoforms of PDE2A and miR-139 (Fig. 5A). Further bioinformatics analysis revealed that the P1 region harbored several Hes1-recognized elements (E-box sites) (Fig. 5B). Hes1 is a downstream molecule of Notch signaling that mediates transcriptional repression by binding E-box sites. Interestingly, Notch1, which is the major receptor of Notch signaling in the brain 33, was identified as a target of miR-139 by our analysis (Fig. 5C-D) and other research studies 34. The mRNA (Fig. 5E) and protein (Fig. 5F) levels of Notch1 were reduced in miR-139-overexpressing glioma cells. In addition, the expression of Notch downstream molecules, Hes and Hey family members, was also repressed after miR-139 overexpression (Fig. 5G). These results indicated that miR-139 obviously reduced the expression of Notch1 and the subsequent Notch signaling activation.

Next, the Notch1-Hes1 axis regulating miR-139 expression was evaluated. A reporter assay revealed that forced activation of Notch signaling could reduce the promoter activity of the wild-type P1 region (Fig. 5H). When E-box site 1 was destroyed, the modulation of Notch signaling in the P1 region disappeared. However, the E-box site 2 mutant did not have this kind of effect (Fig. 5H). Moreover, the ChIP assay also demonstrated that Hes1 recognized and bound with E-box site 1 rather than E-box site 2 (Fig. 5I-J). We next verified that Notch signaling activation obviously reduced PDE2A and miR-139 expression (Fig. 5K). In contrast, when Notch signaling was inhibited by Notch1 siRNAs (Fig. 5L) or the inhibitor GSI (Fig. 5M), the mRNA levels of PDE2A and miR-139 were elevated. Considering that Notch1 is a target of miR-139, we overexpressed miR-139 in glioma cells and found that miR-139 increased the expression of the host gene PDE2A and pre-miR-139, suggesting that miR-139 could amplify its own expression (Fig. 5N-O). The above data indicated that miR-139 could promote its own expression via the miR-139-Notch1/Hes1 feedback circuit.

### OCP-miR-139 and OCP-PDE2A suppressed glioma tumorigenesis by inhibiting stemness

**Figure 6:**
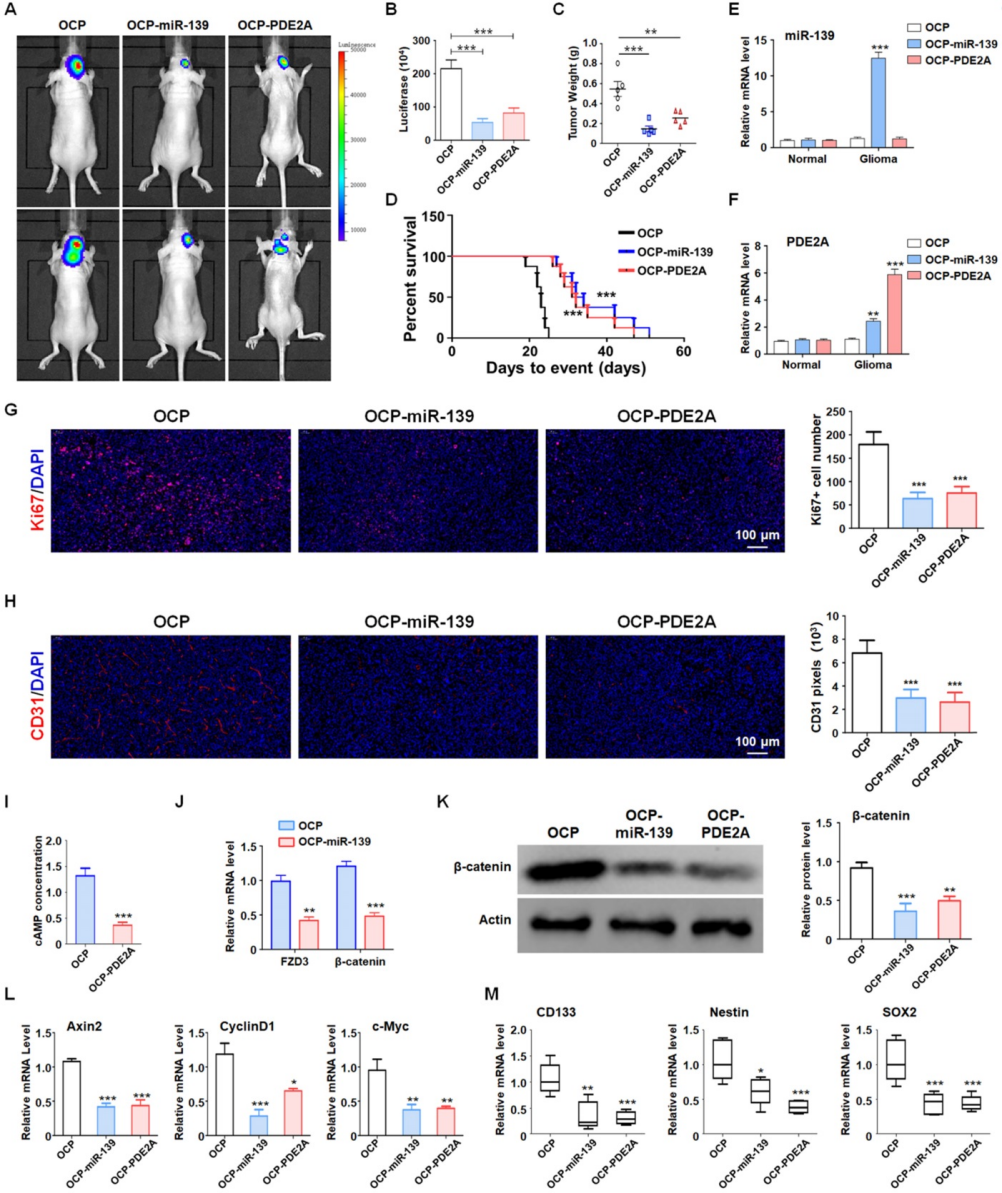
** PDE2A/miR-139 suppressed the tumorigenesis ability of GSCs* in vivo* by inhibiting stemness. (A-B)** Luciferase-modified PD-GSCs were inoculated intracranially into nude mice followed by OCP-miR-139 or OCP-PDE2A administration. Glioma development was evaluated by bioluminescence imaging three weeks after OCP complexes treatment (n = 5). **(C)** The tumor tissues were excised, and the tumor weights were measured after the mice were sacrificed (n = 5). **(D)** The survival time of tumor-bearing mice was monitored for Kaplan-Meier survival curve analysis (n = 8). **(E-F)** The expression of miR-139 and PDE2A was detected in glioma and normal brain tissues (n = 5). **(G)** Ki67 staining was carried out to evaluate the tumor cell proliferation of the different groups (n = 5). **(H)** CD31 staining by immunofluorescence was performed to detect tumor angiogenesis (n = 5). **(I)** The cAMP concentration was determined in Ctrl- and PDE2A-overexpressing glioma tissues (n = 5). **(J)** The expression of FZD3 and β-catenin was detected in Ctrl and miR-139-overexpressing glioma tissues (n = 5). **(K)** The protein levels of β-catenin were determined in gliomas in all groups (n = 5). **(L)** The activation of Wnt/β-catenin signaling was evaluated by detecting expression of downstream genes in gliomas in all three groups (n = 5). **(M)** The expression of stemness markers was determined in tumor tissues (n = 5). Bars, means ± SEM; *, P < 0.05; **, P < 0.01; ***, P < 0.001.

Although PDE2A and miR-139 strongly repressed the stemness of GSCs, their effect on GSC tumorigenesis *in vivo* should be further validated. The luciferase-modified PD1-GSCs were inoculated intracranially into nude mice. And the overexpression plasmid of miR-139 and PDE2A were packaged into OCP, which could deliver DNA fragment into glioma tissue specifically. Then the tumor-bearing mice was injected with OCP, OCP-miR-139 or OCP-PDE2A once per week. Three weeks after the administration, glioma growth was evaluated by bioluminescence imaging and tumor weights. The results indicated that OCP-miR-139 or OCP-PDE2A administration obviously suppressed glioma progression (Fig. 6A-C). Meanwhile, the Kaplan-Meier survival analysis suggested that OCP-miR-139 or OCP-PDE2A increased the survival time of tumor-bearing mice (Fig. 6D). To validate the targeted overexpression of OCP complex, the glioma tissue and normal brain tissue were excised for mRNA detection, which demonstrated that OCP complex only elevated the expression level of genes in glioma specifically (Fig. 6E and 6F). Besides, OCP-miR-139 or OCP-PDE2A treatment also repressed glioma cell proliferation (Fig. 6G) and tumor angiogenesis (Fig. 6H) by histologic staining.

**Figure 7:**
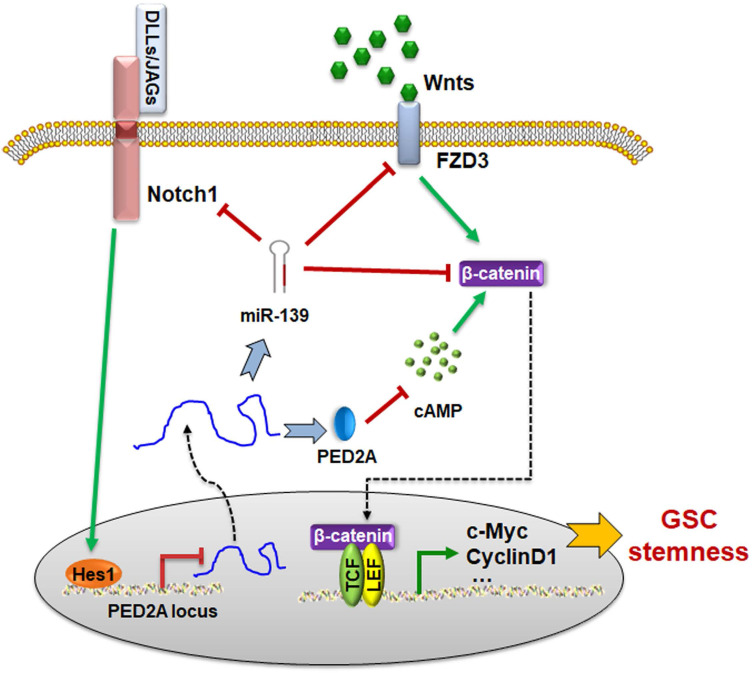
The schematic diagram of the PDE2A/miR-139-Notch1 feedback circuit suppressing the stemness maintenance of GSCs by inhibiting Wnt/β-catenin signaling.

Next, we examined the cAMP concentration and target gene levels in PDE2A- and miR-139-overexpressing gliomas. The results indicated that OCP-PDE2A reduced cAMP concentration in gliomas (Fig. 6I) and that OCP-miR-139 decreased the expression of FZD3 and β-catenin (Fig. 6J). Additionally, the activation of Wnt/β-catenin signaling was suppressed in both PDE2A- and miR-139-overexpressing gliomas (Fig. 6K-L). The expression of stem cell-associated molecules, including CD133, Nestin and SOX2, was downregulated by OCP-miR-139 or OCP-PDE2A (Fig. 6M). The above data demonstrated that both PDE2A and miR-139 presented strong tumor repression functions *in vivo* by inhibiting Wnt/β-catenin signaling. In summary, our current study first shows that miR-139 amplified its own expression and effect and those of its host gene by the miR-139-Notch1/Hes1 feedback loop. Moreover, miR-139 and the host gene PDE2A decreased the stemness maintenance and tumorigenesis of GSCs by cooperatively modulating Wnt/β-catenin signaling (Fig. 7).

## Discussion

GBMs have remained the most lethal malignancies in the CNS, with a survival time of less than 15 months after diagnosis. Despite recent advances in our understanding of GBM pathogenesis via genetically engineered mouse models 35 and global genetic analyses 36, few strategies or methods have been proposed for early diagnosis and treatment. The barriers that limit GBM treatment, including heterogeneity, radiation and chemotherapy resistance and recurrence, have been mainly attributed to the existence of GSCs. Therefore, it is important to elucidate the intrinsic regulators driving GSC generation or function. miRNAs modulate cell fate determination and tissue development based on multiple targets 13. Each miRNA regulates distinct downstream molecules, and one gene could be repressed by different miRNAs, which enhances the effectiveness and robustness of the regulation mediated by miRNAs 11, 12. Many miRNAs act as crucial proto-oncogenes or tumor suppressors in tumor progression 37. In our previous study, miR-139 was shown to repress glioma cell invasion and proliferation and induce apoptosis 21. Additionally, miR-139 prominently enhanced the antitumor effect of temozolomide (TMZ) through direct posttranscriptional regulation of Mcl-1 22. However, the effect of miR-139 on cancer stem cells remains unclear.

In this study, we demonstrated that miR-139 expression was obviously elevated in differentiated GSCs and normal glioma cells compared with GSCs. The overexpression of miR-139 promoted stemness loss while reducing the self-renewal of GSCs. PDE2A, which is the host gene of miR-139, shared a strong positive correlation with miR-139 expression in glioma tissues. The ChIP sequence data in the cistrome database showed that miR-139 had no separate promoter in the brain, which indicated that miR-139 expression was associated with PDE2A transcription. It has been reported by our group and others that miR-139 suppressed different target genes to modulate tumor cells 17, 21, 22. In this study, we demonstrated that a receptor and a core transcription factor of Wnt signaling were both targets of miR-139 regulating GSC self-renewal and differentiation. On the other hand, PDE2A belongs to the phosphodiesterase (PDE) family, which is responsible for the hydrolysis of cyclic phosphate to balance the concentration of cAMP and cGMP 27, 38. The cyclic nucleotides cAMP and cGMP are ubiquitous second messengers that modulate a wide array of intracellular processes 39, 40. The expression of PDEs is tissue specific, and PDE2A is abundant in the brain 38, 41. The aberrant expression of PDE2A causes CNS disorders, such as learning and memory dysfunction 42, 43. We first demonstrated that the PDE2A level was reduced in GSCs, leading to cAMP accumulation. cAMP-regulating cascades activate Wnt signaling through GSK3β phosphorylation and β-catenin nuclear translocation. Wnt signaling plays significant roles in the progression of multiple tumors 26, 44-47, and was required for GSC development and self-renewal 26. The deficiency of Wnt/β-catenin signaling repressed glioma progression 48-50. Our results suggested that overexpression of both miR-139 and PDE2A could repress Wnt/β-catenin signaling significantly and reduced the stemness maintenance and tumorigenesis of gliomas.

Our data (Fig. 5) and other publications have demonstrated that Notch1 is another direct target of miR-139. Notably, the expression of PDE2A/miR-139 was also modulated by Notch signaling. The promoter region of PDE2A harbored one effective Hes1 recognition site, which could be negatively regulated by activated Notch1. Further validation demonstrated that the overexpression of miR-139 increased the mRNA levels of PDE2A and pre-miR-139. A reporter assay and ChIP analysis indicated that Notch-Hes1 could directly repress PDE2A/miR-139 transcription through a binding site in the promoter region (P1). These data suggested that miR-139 could promote its own expression via the miR-139-Notch1/Hes1 double negative feedback loop.

However, the blood brain barrier (BBB) and tumor cells specificity were the principal problems of medicine for glioma therapy. It is urgent to develop a deliver approach to carry DNA or plasmid across the BBB and afterwards target gliomas. The OX26/CTX-conjugated PEGylated liposome (OCP) was able to resolve these problems 23. PDE2A and miR-139 carried by OCP were successfully overexpressed the target genes in gliomas without influence normal brain tissues. Expectedly, OCP-PDE2A and OCP-miR-139 repressed the stemness of GSCs and decelerated glioma progression by inhibiting Wnt/β-catenin signaling activation. Our study showed that the primary transcript of PDE2A/miR-139 inhibited Wnt/β-catenin signaling to modulate GSC stemness and tumorigenesis. The modulation axis could be a potential therapeutic target for gliomas by OCP medicine deliver system.

## Supplementary Material

Supplementary figures.

## Abbreviations

Δβ-cat: activated β-catenin
BBB: blood brain barrier
ChIP: chromatin immunoprecipitation
CNS: central nervous system
DAPI: 4',6-diamidino-2-phenylindole
DMEM: Dulbecco's modified Eagle's medium
FBS: fetal bovine serum
GBM: glioblastomas
GSC: glioma stem cells
GSI: gamma secretase inhibitor
miRNA: MicroRNAs
OCP: OX26/CTX-PL
ORF: open reading frames
PDE: phosphodiesterase
PD-G: patient-derived glioma
PD-GSC: patient-derived glioma stem-like cell
