# Supplementary material for: miR-139/PDE2A-Notch1 feedback circuit represses stemness of gliomas by inhibiting Wnt/β-catenin signaling
Source: Int J Biol Sci. 2021 Aug 12;17(13):3508–21. doi: 10.7150/ijbs.62858 (PMC8416740; doi:10.7150/ijbs.62858)
Supplement: Supplementary file 1 — Supplementary figures. [file ijbsv17p3508s1.pdf]

Supplementary Figures

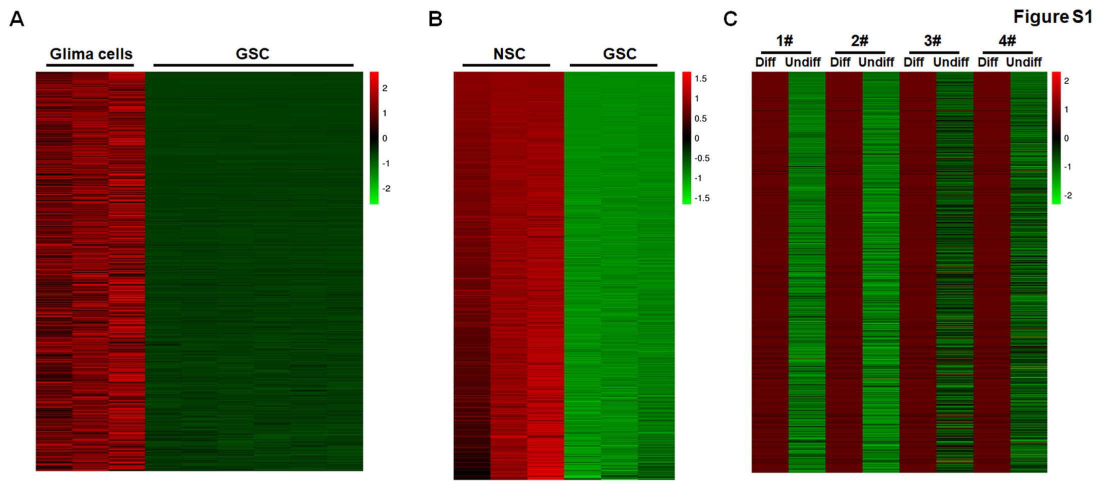

Figure S1. The genes downregulated in GSCs were analyzed in different public datasets. (A-C) Publicly available differential mRNA expression profiles between GSCs and NSCs (GSE41033; n = 3) (A), GSCs and glioma cells (GSE124145; glioma cells, n = 3; GSCs, n = 6) (B) and GSCs and differentiated GSCs (GSE68343; n = 4) (C) were analyzed.

Figure S2

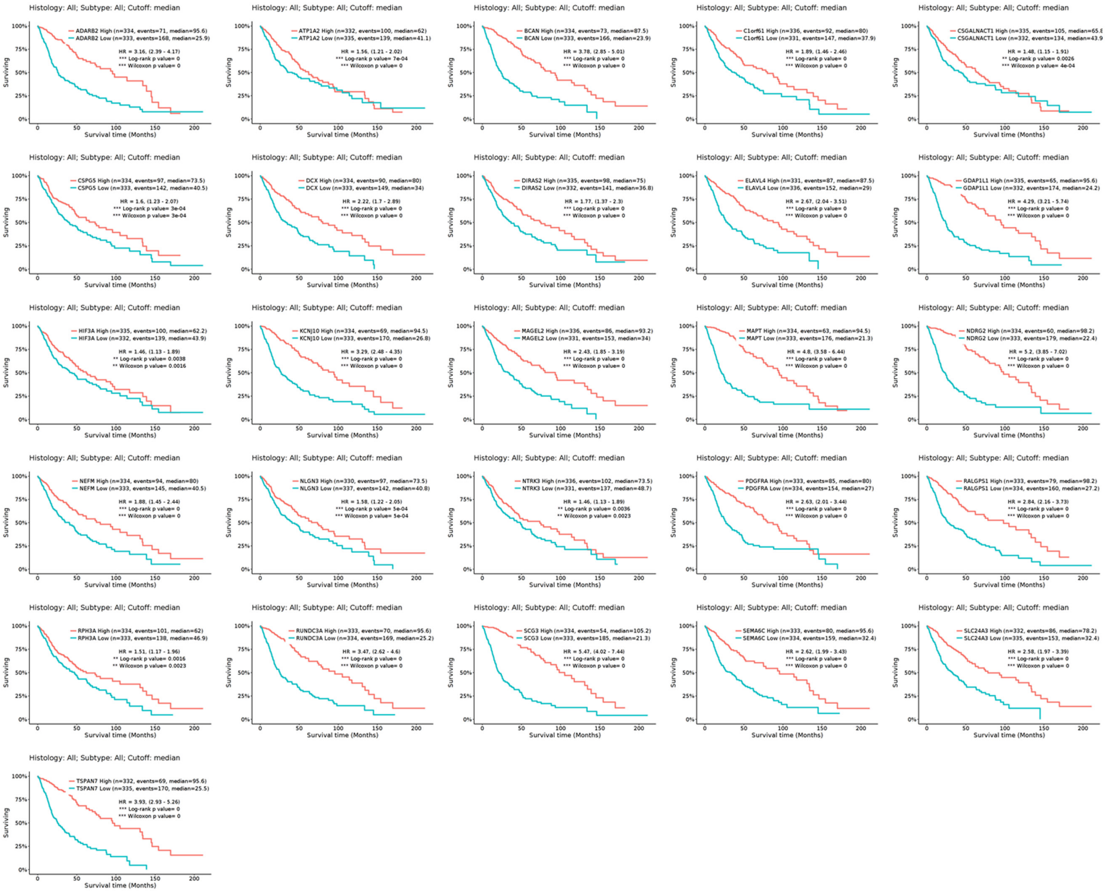

Figure S2. The higher expression of candidate GSC suppressors predicted a better prognosis of glioma patients from the TCGA database. Kaplan-Meier survival analysis of glioma patients is represented according to the expression levels of the 36 common GSC-downregulated genes from different public data. Twenty-six molecules were chosen as candidate GSC suppressors.

Figure S3

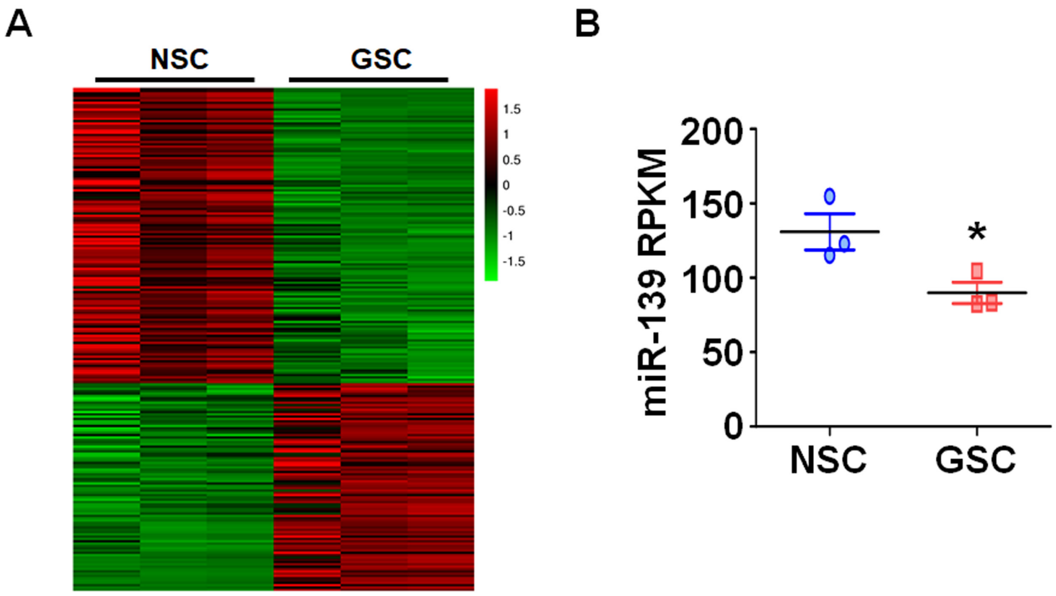

Figure S3. The expression of miR-139 decreased in GSCs compared with NSCs. (A) The miRNA expression levels between NSCs and GSCs were compared in the public dataset GSE41033 (n = 3). (B) The expression alteration of miR-139 between NSCs and GSCs is presented (n = 3). Bars, means ± SEM; *, $P < 0.05$.

Figure S4

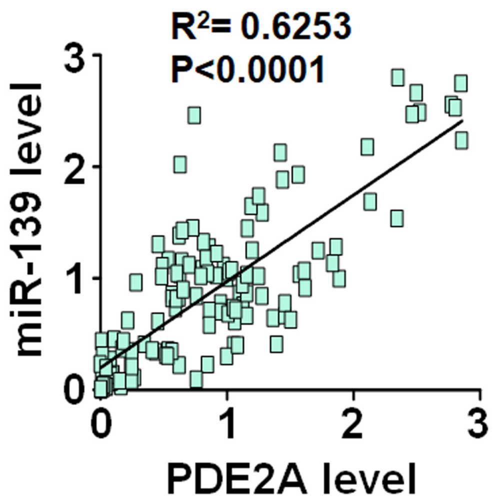

Figure S4. The mRNA expression levels of PDE2A and miR-139 were consistent in glioma tissues. The mRNA expression levels of PDE2A and miR-139 were determined in glioma tissues (n = 125).

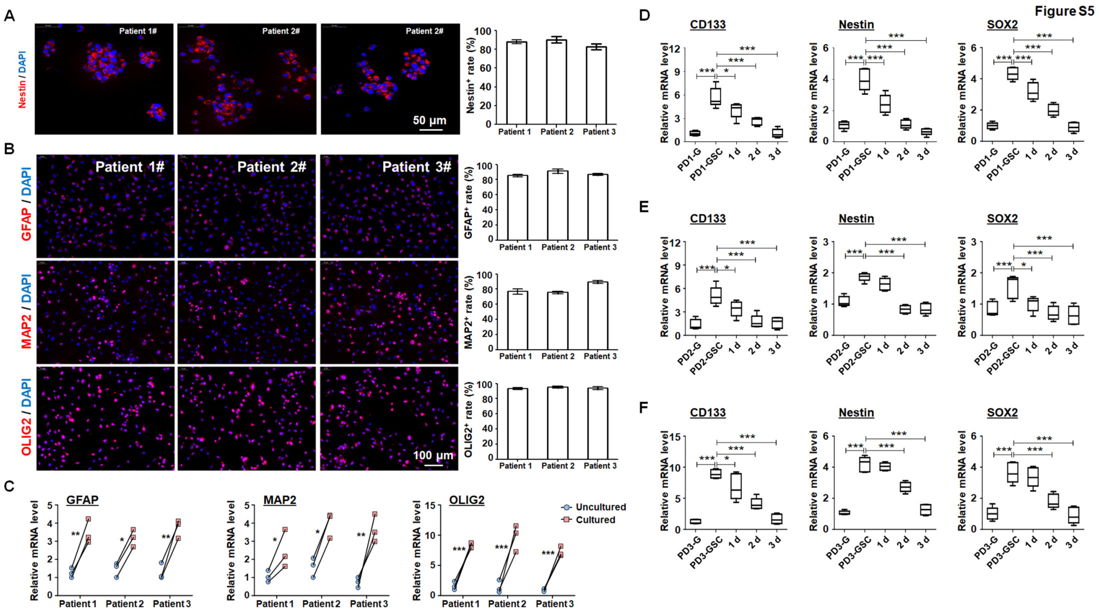

Figure S5. The expression levels of PDE2A and miR-139 decreased in patient-derived GSCs. (A) Patient-derived glioma cells (PD-G) were isolated from three GBM patients and stimulated to transform into glioma stem cells (PD-GSCs). The PD-GSCs were identified by Nestin staining (n = 3). (B) PD-GSCs were cultured in complete medium containing FBS for pluripotent differentiation determination. The differentiated cells were stained by GFAP, MAP2 and OLIG2 (n = 3). (C) The expression of GFAP, MAP2 and OLIG2 was detected in PD-GSCs with or without culture. (D-F) PD-GSCs were cultured in complete medium for differentiation. Stemness marker expression was determined in PD-G, maintained PD-GSCs and differentiated PD-GSCs from the three patients (n = 6). Bars, means ± SEM; *, $P < 0.05$; **, $P < 0.01$; ***, $P < 0.001$.

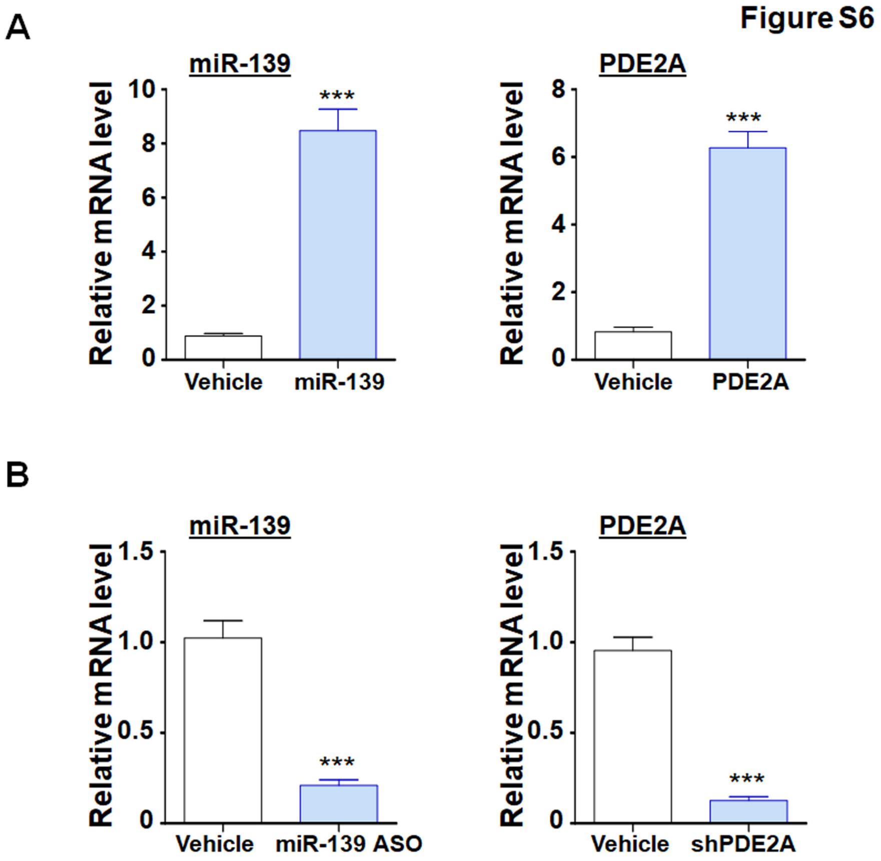

Figure S6. The overexpression or repression efficiency of miR-139 and PDE2A was determined in patient-derived GSCs. (A) PD-Gs were isolated from GBM patient 1# and overexpressed miR-139 or PDE2A by lentivirus. The overexpression efficiency was detected (n = 5). (B) MiR-139 or PDE2A was knock down by lentivirus in PD-Gs isolated from GBM patient 1# and the repression efficiency was detected (n = 5). Bars, means ± SEM; ***, P < 0.001.

Figure S7

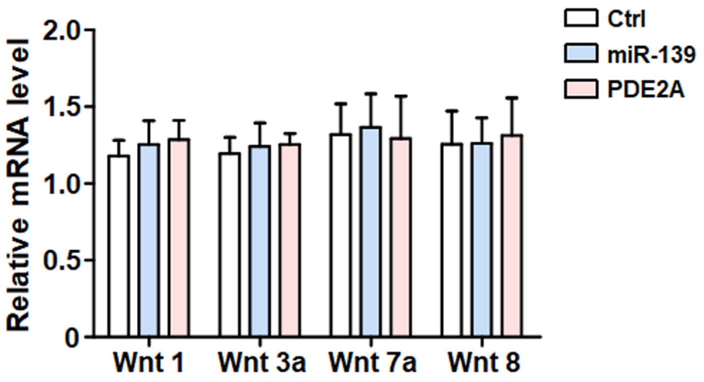

Figure S7. The expression of Wnt ligands was determined in glioma cells after overexpression of miR-139 or PDE2A (n = 5). Bars, means ± SEM.
